# Access to interpreting services in England: secondary analysis of national data

**DOI:** 10.1186/1471-2458-9-12

**Published:** 2009-01-12

**Authors:** Paramjit S Gill, Aparna Shankar, Terry Quirke, Nick Freemantle

**Affiliations:** 1 School of Health and Population Sciences, University of Birmingham, Birmingham, UK

## Abstract

**Background:**

Overcoming language barriers to health care is a global challenge. There is great linguistic diversity in the major cities in the UK with more than 300 languages, excluding dialects, spoken by children in London alone. However, there is dearth of data on the number of non-English speakers for planning effective interpreting services. The aim was to estimate the number of people requiring language support amongst the minority ethnic communities in England.

**Methods:**

Secondary analysis of national representative sample of subjects recruited to the Health Surveys for England 1999 and 2004.

**Results:**

298,432 individuals from the four main minority ethnic communities (Indian, Pakistani, Bangladeshi and Chinese) who may be unable to communicate effectively with a health professional. This represents 2,520,885 general practice consultations per year where interpreting services might be required.

**Conclusion:**

Effective interpreting services are required to improve access and health outcomes of non-English speakers and thereby facilitate a reduction in health inequalities.

## Background

Overcoming language barriers to health care is a global challenge. [1-3] In the US over 24 million residents are unable to speak English fluently, with over 55 million residents speaking a language other than English. [4] In urban Australia language services are required in up to 100 different languages reflecting enormous linguistic diversity. [5] The UK is a diverse society with 7.9% of the population from Black and other minority ethnic groups.[6] This is a heterogeneous group with different migration and settlement patterns, culture, religion, and languages spoken. Recent research identifying more than 300 languages, excluding dialects, spoken by children at home indicates that London may be the most linguistically diverse city in the world. [7]

It is obvious that high-quality medical care requires effective communication between patient and health professional. [8] The complexity of linguistic diversity is illustrated with a report that found that many doctors working in primary care in the UK are themselves not native English speakers and communicate with their patients, originally from the Indian subcontinent, in one of a range of Asian languages.[9] An obvious benefit of this is the shared understanding and knowledge of health beliefs and expectations from health care professionals. [10] However, when faced with English-speaking health professionals, patients with no functional English will require interpreting services. This may often include informal interpreters such as family members [11] although this can be problematic when faced with embarrassing issues or when the informal interpreter's language skills are poor. [11] While even good quality professional interpreting will not completely remove the language barrier, effective communication can be achieved and has been shown to lead to improved care [12], comparable to that received by English-speaking patients. [13]

Currently no national data exist on the number of non-English speakers in the UK and there is a need to estimate this to plan effective interpreting services. The aim of this study was to estimate the number of people requiring language support amongst the minority ethnic communities.

## Methods

The present analysis combined data from the Health Survey for England 1999 [14] and 2004 [15]. Both surveys included boost samples from ethnic minority groups and the current analysis focussed on four minority ethnic groups (Indians, Pakistanis, Bangladeshis and Chinese). The surveys use a multi-stage, stratified probability sampling design. Primary sampling units (postcode sector in 1999 and census wards in 2004) were stratified based on the proportion of resident individuals from ethnic minorities. The probability of each unit being selected was proportional to the number of addresses within the unit. Screening of addresses and focussed enumeration (in areas where there was a smaller proportion of minority ethnic residents) was carried out to identify individuals for inclusion in the survey. In 2004, in addition to the minority ethnic boost sample, a further Chinese boost sample was obtained by screening the electoral register for individuals with 'Chinese-sounding' surnames. In 1999, the Chinese sample was recruited solely by re-contacting individuals who participated in the 1998 Health and Lifestyles of the Chinese Population in England survey. [16] Further information on the methodology used in the surveys is available elsewhere. [14,15]

Participants were asked how well they spoke English (self-report). Response options included 'very well', 'fairly well', 'slightly' and 'not at all'. The proportion of participants who responded 'slightly' or 'not at all' was calculated for participants aged 16 years and above by age category (16–34, 35–54 and 55+), ethnic group and sex. The analyses were weighted to correct for differing selection probabilities. This proportion was applied to data for England from the Census 2001 [17] to provide an estimate of the number of individuals in the population from these groups who are unable to converse in English. This was then multiplied by the annual contact rate for GP consultations based on the HSE 1999 [14] to provide an estimate of the number of GP consultations per year for these minority groups where interpreting services are likely to be required.

## Results

Eight thousand and forty one participants were included in this analysis (47.4% male). We estimated that there are 298,432 individuals from these ethnic groups in the population who are unable to converse in English (Tables 1 and 2). The proportion of individuals unable to speak English increases with age and fewer women speak English. Based on average annual contact rate by sex, age group and ethnicity, we estimated that annually 2,520,885 general practitioner consultations for individuals from these ethnic groups are likely to require interpreting services. The issue is particularly important for women and older individuals as they also report higher consultation rates.

**Table 1:** Percentage of male individuals who speak little or no English, estimates of the number in England and the number of annual GP consultations by age and ethnic group in 1999 and 2004

	*% of individuals who speak little or no English*	*Estimated number in population*	*Estimated number of GP consultations/year*
Indian			
16–34	2.9 (1.7–4.7)	4998	14993
35–54	6.0 (4.3–8.2)	8823	50293
55+	20.9 (16.6–25.8)	14923	137292
Pakistani			
16–34	9.8 (7.4–12.7)	12957	24618
35–54	15.1 (11.5–19.3)	10020	66131
55+	35.3 (27.5–43.8)	11740	123274
Bangladeshi			
16–34	14.8 (11.3–18.9)	7573	22718
35–54	36.2 (29.5–43.3)	7748	66635
55+	65.6 (56.5–73.8)	8248	122894
Chinese			
16–34	9.3 (5.5–14.5)	4172	5423
35–54	26.0 (19.8–33.0)	7628	21357
55+	57.1 (46.1–67.6)	6457	32933

**Table 2:** Percentage of female individuals who speak little or no English, estimates of the number in England and the number of annual GP consultations by age and ethnic group in 1999 and 2004

	*% of individuals who speak little or no English*	*Estimated number in population*	*Estimated number of GP consultations/year*
Indian			
16–34	5.3 (3.7–7.2)	9442	49098
35–54	14.6 (12.1–17.4)	22136	148311
55+	45.2 (39.5–51.0)	32721	458090
Pakistani			
16–34	15.8 (13.0–18.8)	20999	151189
35–54	40.1 (35.0–45.5)	26743	243358
55+	67.8 (58.4–763)	18817	319893
Bangladeshi			
16–34	31.2 (27.1–35.6)	17134	80530
35–54	75.9 (68.9–82.0)	15988	174265
55+	91.1 (82.5–96.4)	7706	77060
Chinese			
16–34	6.3 (3.1–11.0)	2868	5162
35–54	29.8 (24.4–35.6)	11041	57416
55+	61.2 (49.2–72.2)	7550	67952

## Discussion

This study shows that nearly 300,000 adults from the four main ethnic communities in England and Wales have no functional English to communicate with their health professional. Even though these communities have been resident in the UK for over 30 years, the differences by ethnic group partly reflect the different migration patterns. [6] An inability to communicate in English can create barriers, misunderstandings and misconceptions in patient-health professional relationships [8] and patients themselves repeatedly highlight ineffective communication as cause of unsatisfactory experiences of health services. [18] Further, patients are unlikely to be able to participate in and contribute fully to their local community. [19] Despite this, fluency in English is not routinely documented as part of ethnic monitoring. [20]

Estimates of the number of individuals unable to converse in English vary widely, from 400,000 to 1.2 million. [20] With recent migration of other communities this number is likely to be even higher. [21] Further, as our estimates are based on 2001 Census data, ethnic group populations have grown and the Office of National Statistics has now produced experimental population estimates by ethnic group. [22] Our estimate is slightly lower than that reported by Carr-Hill et al [19] whose sample was smaller with 925 Punjabi, Bengali and Chinese speakers and 173 subjects from 4 refugee groups.

There is a great need for effective interpreting services across the country and provision is patchy with access restricted to health professionals. Some of this interpreting is provided by informal interpreters such as family members [11] and general practitioners. [9] However, the latter are due to retire within the next few years and further increasing demand for interpreting services. [23]

It is estimated that the additional costs (English Language Difficulties Adjustment) of providing medical services to patients who do not speak English is £29/person. [6] This adjustment grossly underestimates the number of non-English speakers and fails to highlight the scale and distribution of this population. In our present study, we used consultation rates from the HSE, which are self-reported and may be an underestimate [6,24,25]. There is a need to map the distribution of non-English speakers, hence inclusion of language spoken as part of the new Census dataset. [6,20] This will then ensure the appropriate and effective provision of interpreting services within the UK and through this, improved access and health outcomes for migrants with subsequent reduction in health inequalities. [26]

## Conclusion

We have highlighted the unmet need for interpreting services within four minority ethnic communities and with increased international migration; demand for effective interpreting will continue to rise.

## Competing interests

The authors declare that they have no competing interests.

## Authors' contributions

PG initiated the study, supervised its conduct and analysis and drafted the paper with all authors. AS and NF produced the analysis plan. AS and TQ did the statistical analysis. PG is the guarantor.

## Pre-publication history

The pre-publication history for this paper can be accessed here:


